# Learning Distinct Chemical Labels of Nestmates in Ants

**DOI:** 10.3389/fnbeh.2018.00191

**Published:** 2018-08-29

**Authors:** Stefanie Neupert, Manuel Hornung, Jocelyn Grenwille Millar, Christoph Johannes Kleineidam

**Affiliations:** ^1^Department of Neurobiology/Zoology, Universität Konstanz, Konstanz, Germany; ^2^Department of Entomology, University of Riverside, Riverside, CA, United States

**Keywords:** pattern recognition, olfaction, label-template matching, cuticular hydrocarbons, synthetic odor processing, social insects, *Camponotus floridanus*

## Abstract

Colony coherence is essential for eusocial insects because it supports the inclusive fitness of colony members. Ants quickly and reliably recognize who belongs to the colony (nestmates) and who is an outsider (non-nestmates) based on chemical recognition cues (cuticular hydrocarbons: CHCs) which as a whole constitute a chemical label. The process of nestmate recognition often is described as matching a neural template with the label. In this study, we tested the prevailing view that ants use commonalities in the colony odor that are present in the CHC profile of all individuals of a colony or whether different CHC profiles are learned independently. We created and manipulated sub-colonies by adding one or two different hydrocarbons that were not present in the original colony odor of our *Camponotus floridanus* colony and later tested workers of the sub-colonies in one-on-one encounters for aggressive responses. We found that workers adjust their nestmate recognition by learning novel, manipulated CHC profiles, but still accept workers with the previous CHC profile. Workers from a sub-colony with two additional components showed aggression against workers with only one of the two components added to their CHC profile. Thus, additional components as well as the lack of a component can alter a label as “non-nestmate.” Our results suggest that ants have multiple-templates to recognize nestmates carrying distinct labels. This finding is in contrast to what previously has been proposed, i.e., a widening of the acceptance range of one template. We conclude that nestmate recognition in ants is a partitioned (multiple-template) process of the olfactory system that allows discrimination and categorization of nestmates by differences in their CHC profiles. Our findings have strong implications for our understanding of the underlying mechanisms of colony coherence and task allocation because they illustrate the importance of individual experience and task associated differences in the CHC profiles that can be instructive for the organization of insect societies.

## Introduction

In eusocial insects, the ability to discriminate between members from one’s own colony (nestmates) and members from a foreign colony (non-nestmates) is of fundamental importance for colony coherence, and ultimately benefits colony fitness (Hölldobler and Wilson, 1990). Discrimination between nestmates and non-nestmates prevents the colony’s resources (e.g., food storage, brood) from being exploited by competing conspecific and heterospecific colonies, predators and parasites. In ants, the discrimination between nestmates and non-nestmates relies on olfaction and is based on mixtures of low-volatile chemical components on the ants’ exoskeleton (cuticular hydrocarbons: CHCs; Lahav et al., 1999). The primary functions of CHCs are to prevent desiccation (Lockey, 1988) and protect against infections. Secondarily, CHCs were exploited as recognition cues for colony membership. The composition of CHCs is species-specific (Martin S. and Drijfhout F., 2009) and genetically determined, but individuals’ CHC profiles are additionally shaped by diet and nest material (Jutsum et al., 1979; Vander Meer et al., 1989). Frequent exchange of CHCs between nestmates through trophallaxis and allogrooming (Lenoir et al., 2001) results in a uniformed, colony-specific chemical signature (Crozier and Dix, 1979). This signature commonly is called the colony odor and consists of all CHC profiles carried by individuals of one colony. The colony odors of neighboring, conspecific colonies generally differ only in the quantitative ratios of CHCs. Because diet, nest material and colony composition may change, the colony odor is not constant but varies over time (Vander Meer et al., 1989). Importantly, CHC profiles among individual workers of a colony are not as uniform as previously assumed. Workers have task-specific CHC profiles because task performance, e.g., nest building, brood tending, or foraging outside the nest influence the CHC profiles (Wagner et al., 1998; Kaib et al., 2000). Furthermore, the mixing of CHCs between individuals of a colony is not complete because workers within a task-group encounter and interact with each other more frequently than between task-groups (Sendova-Franks and Franks, 1995; Mersch et al., 2013; Pamminger et al., 2014; Tschinkel and Hanley, 2017). Based on these systematic differences in CHC profiles, workers are able to discriminate between nestmates performing different tasks (Bonavita-Cougourdan et al., 1993; Greene and Gordon, 2003).

Although CHC profiles vary within a colony, aggression among nestmates is rare and nestmates are discriminated from conspecific non-nestmates rapidly and with high reliability—even though conspecific non-nestmates can have CHC profiles which are qualitatively identical and only differ in component ratios (Stroeymeyt et al., 2010). It is not known how workers discriminate between nestmates and non-nestmates based on the ratios of CHCs. To understand the neuronal and behavioral mechanisms of nestmate recognition, we need to examine all parts of the recognition system from CHC profiles to behavioral reactions. This involves analyzing how information contained in the CHC profile is detected and processed by the nervous system, how “nestmate” and “non-nestmate” is encoded in the brain, how this influences decision making, and eventually results in an observable behavioral outcome during encounters.

Different models and possible strategies to discriminate between nestmates and non-nestmates have been proposed. Based on the phenotype matching model (Holmes and Sherman, 1983), each individual carries both recognition cues of its own CHC profile (a label) and a neural representation (a template) of its own colony odor (Lacy and Sherman, 1983). In theory, the label from an encountered individual is compared with the “own” template, denoted as a label-template matching process. In its most simple form, individuals are hypothesized to be anosmic to their own colony odor and so colony-specific labels are not perceived, and all labels causing a salient perception are considered as belonging to non-nestmates. Although this idea has been proposed prominently (Ozaki et al., 2005), it fails to explain how workers can discriminate between nestmates of different task-groups (Bonavita-Cougourdan et al., 1993; Greene and Gordon, 2003) and subsequent studies have shown that workers are not anosmic to their own colony odor (Brandstaetter and Kleineidam, 2011; Brandstaetter et al., 2011; Sharma et al., 2015).

The most widely accepted model for nestmate recognition considers the frequently experienced labels of nestmates during early adulthood as being instructive for the formation of a template (Carlin and Hölldobler, 1983; Morel and Blum, 1988; Errard, 1994). This learning process allows individuals to recognize nestmates and to discriminate between “we” vs. “others.” Because the colony odor can change over time, individuals need to adjust their template accordingly. Manipulation of the colony odor leads to acceptance of both the current colony odor as well as the previous colony odor, and for a single template this would require a widening of the acceptance range (Leonhardt et al., 2007; Guerrieri et al., 2009; Bos and d’Ettorre, 2012; Bos, 2014).

Because diverse labels can be employed for recognizing nestmates (Fielde, 1903; Errard, 1994), it is puzzling how workers can still achieve high reliability in nestmate recognition by using a single template. The specificity range of this neural template has to be broad enough to cover the variety of different labels that are present within the colony, and at the same time exclude labels of non-nestmates (Reeve, 1989).

As an alternative to a single, unifying-template, we hypothesize that workers use multiple templates, each with its own specificity range, for recognizing nestmates. In this case, chemically distinct labels are recognized with different templates, which possibly are attributed with further information about the label carrier. Labels identified from distinct templates may be perceptually kept separately or are generalized as all belonging to “nestmates.” Such a recognition system with multiple templates is comparable to associative learning, analogous to honeybees learning the association between flowers and floral odorants, where flowers provide a species-specific chemical pattern and many different types of floral odorant profiles can be learned (Bitterman et al., 1983; Gerber et al., 1996). The difference between these two nestmate recognition hypotheses is that in the one-template nestmate recognition system, commonalities in the CHC profiles of the colony odor are used for nestmate recognition, whereas in the multiple-template nestmate recognition system chemically distinct CHC profiles within a colony are recognized independently of each other and categorized as belonging to nestmates.

**Figure 1:**
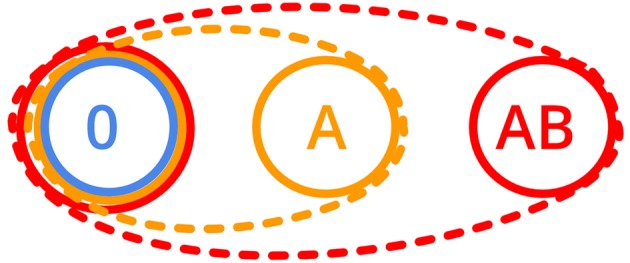
Schematic acceptance ranges as ovals (dashed lines) for the one-template and as circles (solid lines) for the multiple-template nestmate recognition system (0: no additional components, A: one additional, AB: two additional components added to the cuticular hydrocarbon, CHC profile).

To investigate these two possible mechanisms of nestmate recognition, we systematically manipulated the CHC profiles of workers originating from the same colony by adding none, one or two hydrocarbon(s) and tested if they still accept each other as nestmates. Based on a one-template nestmate recognition system, we predict that workers which incorporated two additional hydrocarbons into their CHC profile also accept individuals that have only one hydrocarbon added (Figure 1). In contrast, if workers with two additional hydrocarbons do not accept individuals with only one of the hydrocarbons added, the idea of a multiple-template nestmate recognition system is supported.

## Materials and Methods

For all experiments, we used adult workers of the species *Camponotus floridanus* (Buckley, 1866) from a mature colony that was collected at Sugerloaf Shores in Florida by A. Endler in July 2003. Collection of founding queens for laboratory colonies conformed to the laws of the United States of America effective at the time of collection. Colonies of this species have one mated queen (monogynous) which mates with only one male (monoandrous; Gadau et al., 1996) resulting in high genetic homogeneity within a colony compared to polygynous/polyandrous species. In the laboratory, the queen-right colony was kept in an artificial plaster nest at a constant temperature of 25°C and about 40%–50% humidity with a 12:12 h L/D photo period. The colony was fed with honey water and dead locusts twice a week and was provided with water *ad libitum*. The experiments and protocols performed comply with the “*Guide for the Care and Use of Laboratory Animals* of the National Institutes of Health published by National Academic Press (1996).” The protocol was approved by the “Regierungspräsidium Freiburg” in accordance with § 8a (TierSchG) and the current laws of the Federal Republic of Germany (“Tierschutzgesetz”).

### Manipulating Colony Profiles of Sub-Colonies

From the mature colony (source colony), we created four different sub-colonies maintained in separate plaster nests. Each sub-colony received a different treatment by adding either no, one, or two synthetic hydrocarbons to the colony odors of the sub-colonies. For manipulation, we used the following hydrocarbons, which were not present in the original CHC profile of *C. floridanus*: the linear alkane triacontane (C30, Sigma-Aldrich) and hereafter referred to as hydrocarbon A, was added to two sub-colonies (sub-colony A and sub-colony AB). The branched alkane 5-methylheptacosane (5-MeC27), synthesized for this study and hereafter referred to as hydrocarbon B, was added together with A to sub-colony AB. The branched alkane 11-methylheptacosane (11-MeC27), synthesized for this study, hereafter referred to as hydrocarbon C, was added to sub-colony C. One sub-colony was sham-treated as the control (sub-colony 0).

We used a two-step approach to manipulate the CHC profiles. In the first step, we induced an immediate change of the CHC profiles of all workers before transferring them to the respective sub-colonies. In the second step, we counteracted the diminishing of introduced hydrocarbons from the workers’ cuticles by providing hydrocarbon-coated granules at the nest entrance and in the food.

For the first manipulation step, we coated the inner wall of an Erlenmeyer flask (25 ml) with the corresponding hydrocarbon(s) by adding 3 mg of hydrocarbon A (sub-colony A) or 3 mg of hydrocarbon A and 3 mg of hydrocarbon B (sub-colony AB) or 6 mg of hydrocarbon C (sub-colony C), and 6 ml of pentane to the flask. We added a relatively large amount of hydrocarbon C to sub-colony C in order to induce a strong change in the CHC profiles of one sub-colony which should be recognized with a high probability as unfamiliar and belonging to non-nestmates.

The flask was gently shaken and briefly heated to ~60°C on a hot plate to evaporate the solvent, and then cooled to room temperature. We randomly selected five workers from the source colony, cooled them on ice and transferred them to the coated flask. The workers were gently swirled in the flask for 10–15 s and then transferred to a new plaster nest. This was repeated until 100–150 workers had been treated. Workers for sub-colony 0 received the same treatment, except no hydrocarbon was added to the pentane (sham-treatment).

For the second manipulation step, we coated 600 mg of granules (superabsorbent polymers, poly-acrylic acid sodium salt, Stockosorb^®^500) with 6 ml of pentane and 3 mg of hydrocarbon A (sub-colony A) or 3 mg of hydrocarbon A and 3 mg of hydrocarbon B (sub-colony B), or 6 mg of hydrocarbon C (sub-colony C) in a beaker. The beaker was swirled and then put on a hot plate at 60°C to evaporate the pentane. The coated granules then were transferred to a glass tube and small glass vials. We connected the glass tube (diameter: 17 mm; length: 100 mm) to the nest (approx. 100 mm × 80 mm), which served as a long entrance area. Workers entering and leaving the nest passively took up some hydrocarbons from contact with the granules. We added honey water or water to the glass vials and provided them to the sub-colonies in order to facilitate hydrocarbon uptake while feeding. After manipulation, sub-colony 0, A and AB contained approximately 150 adult workers each, and colony C had approximately 100 workers. We provided brood (eggs, larvae and pupae) to each of the sub-colonies to maintain their natural social structure.

Workers in the sub-colonies that were tested later in behavioral experiments were all familiar with the previous, original CHC profile and in the manipulated sub-colonies workers also became familiar with a novel CHC profile. Thus, we created workers with different experience, being familiar with one or two different CHC profiles and unfamiliar with three or two CHC profiles, for sham-treated and manipulated sub-colonies, respectively.

### Behavioral Assay

Starting 1 day after manipulations, we performed one-on-one encounters in small Petri dishes between two workers, either from the same or from different sub-colonies. Because social isolation of workers reduces the propensity for aggression against non-nestmates (Kleineidam et al., 2017), we first transferred one of the workers (stimulus worker) into the Petri dish, and after 1 h, we started the experiment by adding the second worker (focal worker) to the dish. We scored the behavior of the focal worker during the first interaction with the stimulus worker as either being “aggressive” when the focal worker showed one of the behaviors: widely opened mandibles, snapping, body raising or gaster flexing, all of which are typical aggressive behaviors for this species (Brandstaetter et al., 2008). Focal workers which exhibited none of these behaviors were classified as being “non-aggressive.”

We inferred nestmate recognition and discrimination based on the focal workers’ behavioral responses. In cases, where focal workers did not respond with aggression, we categorized this as recognition and possible acceptance as nestmates. In cases where workers from a sub-colony show low probability of aggression against stimulus workers from different sub-colonies, either their CHC profiles are not discriminated, or they are discriminated but without a behavioral readout by the observer. Thus, the workers’ nestmate recognition, as categorized by the observer, either is a generalization with the assumption that workers can discriminate a diversity of CHC profiles or the categorization is based on non-discriminable CHC profiles.

Scoring of the behavioral recordings was conducted blind, with the observer not knowing the stimulus worker type. Immediately after the first interaction, focal and stimulus workers were separated either for examination of CHC profiles (described below), or the focal worker was transferred back to its sub-colony. On the four consecutive days after establishing the sub-colonies and manipulating their colony odors, a total of 360 one-on-one encounters were conducted in six sessions with (almost) balanced types of encounters. For each of the 12 possible encounter types, 25–31 repetitions were conducted; the variation in numbers resulted from the observer-blind experimental design. Because some of the focal workers were transferred back to their sub-colonies after an experiment, it is possible that some focal workers were tested more than once against the same sub-colony. Based on a simulation (5,000 times) with the number of focal workers that were not used for the analysis of their CHC profiles, we calculated that no more than 7.5% of all tested focal workers were selected more than once for an encounter against a stimulus worker from the same sub-colony as before.

### Analysis of Behavioral Data

First, we tested whether the time since establishing the sub-colony significantly influenced the probability of aggression in focal workers. We ran a generalized linear model (GLM) with the binary response variable aggression (“aggression”/“no aggression”) and added time (experimental sessions 1–6) as the only explanatory variable. Because the slope estimate was not significant (*p* > 0.05), we did not include time in the final model (model 2).

Second, we tested for differences in the probability of aggression in focal workers against stimulus workers from the different sub-colonies, using a GLM with the binary response variable aggression (“aggression”/“no aggression”), and as explanatory variables the origin of stimulus worker, origin of focal worker, and an interaction of both variables (model 2).

In order to draw inferences about differences in behavior between focal workers from the different sub-colonies, we used a Bayesian framework. We calculated 20,000 values that are random draws from the posterior distribution of the model 2 estimates. We compared the probability of a worker being aggressive towards stimulus workers from their own and from different sub-colonies. We calculated the proportions of simulated values from the posterior distribution that were larger (or smaller) for focal workers encountering stimulus workers from their own sub-colony compared to focal workers encountering stimulus workers from another sub-colony (measure of certainty). We used the 2.5% and the 97.5% quantiles as the lower and upper limits of the 95% credible interval. Statistical analyses were done using R (v3.4.3; R Core Team, 2017), including the package *arm* with the *sim* function to draw random samples from the posterior distribution of model parameters (Gelman and Hill, 2007). Level of significance for the Bayesian statistics was set to >99% of certainty.

### CHC Extractions

To verify that our manipulation of CHC profiles was successful, we collected workers for CHC extractions after aggression tests (always pairs of focal and stimulus workers) and immobilized the workers on ice. Each worker was held in a 1 ml glass vial and completely covered with pentane for 1 h. The vial was shaken gently and then the pentane was transferred to a clean vial with a 200 μl glass insert. The pentane was allowed to evaporate at room temperature and sealed vials then were stored in a freezer at −20°C until analysis.

### Analysis of CHC Profiles

The vials were defrosted to room temperature, 1–3 drops of pentane were added, and the vials were agitated for 1 min with a vortexer. We then concentrated the sample under a constant stream of air to approximately 20 μl. For coupled gas chromatography mass-spectrometry analysis (GC-MS), we manually injected 1 μl of the samples into a GC (Trace GC Ultra coupled to a DSO II mass spectrometer, Thermo Scientific). The CHCs were analyzed on a fused silica column (Optima-5-MS −0.25 μm, 30 m × 0.25 mm, Macherey-Nagel GmbH and Co. KG) with helium as carrier gas (1.2 ml/min). Chromatograms were recorded with Xcalibur software 1.4 SR1 (Thermo Scientific). The GC oven was programmed as follows: (1) 70°C for 1 min; (2) increase at 30°C/min to 200°C; (3) increase at 3°C/min to 290°C; and (4) hold at 290°C for 5 min.

We analyzed the chemical similarity of workers from different sub-colonies by principal component analysis (PCA) based on the normalized peak areas, including the 10 most prominent peaks from the GC-MS analysis plus the signal at the retention times where the three components (A, B, C) were detected when present.

### CHC Identification

We ran a standard solution of linear hydrocarbons ranging from 21–40 carbons, using the same method as for the CHC profiles. We calculated the Kovats index for each peak in the profile. The CHC compounds were identified based on their Kovats indices and their mass spectra, which were compared to entries of known components in the Wiley and NIST libraries.

### Synthesis of Methyl-Branched Hydrocarbons

#### 5-Methylheptacosane

Butyllithium (2.2 M in hexane) was added dropwise to a slurry of docosyltriphenylphosphonium bromide (3.26 g, 5 mmol) in 100 ml dry tetrahydrofuran (THF) in an oven-dried flask under argon until the mixture retained a pink tinge, followed by addition of a further 2.5 ml of butyllithium solution (5.5 mmol). The resulting clear cherry-red solution was stirred 30 min, followed by addition of 2-hexanone (0.50 g, 5 mmol) in 5 ml THF with a syringe pump over 30 min. The resulting mixture was stirred overnight, then quenched by addition of saturated aqueous NH_4_Cl. The mixture was then diluted with water and extracted with hexane. The hexane layer was washed with water and brine, dried over anhydrous Na_2_SO_4_, concentrated, and purified by vacuum flash chromatography on silica gel, eluting with hexane. The resulting semicrystalline product was taken up in 25 ml hexane in a 50 ml flask, 5% Pd on carbon catalyst was added (250 mg), and the flask was flushed with hydrogen, sealed and stirred under hydrogen for 3 h, when GC analysis showed that all the starting material had been consumed. The mixture was filtered through a celite plug, rinsing well with hexane, and after concentration, the resulting solid was recrystallized from 30 ml acetone overnight at 4°C. Filtration yielded the product as white crystals (0.60 g). EI mass spectrum (70 eV; m/z, abundance): 394 (M+, trace), 379 (7), 365 (7), 351 (3), 337 (54), 336 (31), 309 (7), 308 (10), 295 (1), 281 (2), 267 (2), 253 (3), 239 (3), 225 (3), 211 (3), 197 (4), 183 (5), 169 (5), 155 (7), 141 (8), 127 (10), 113 (13), 99 (19), 85 (100), 84 (73), 71 (56), 57 (81), 43 (71).

#### 11-Methylheptacosane

11-Methylheptacosane was made in similar fashion, starting with hexadecyltriphenylphosphonium bromide and 2-dodecanone, with the exception that the final product was recrystallized first from isooctane and then again from acetone at 4°C, yielding white crystals. EI mass spectrum (70 eV; m/z, abundance): 394 (M+, trace), 379 (5), 365 (3), 351 (2), 337 (2), 323 (2), 309 (1), 295 (1), 281 (1), 267 (1), 253 (22), 252 (26), 239 (1), 225 (3), 224 (7), 211 (2), 197 (3), 183 (4), 169 (21), 168 (45), 155 (5), 141 (7), 140 (7), 127 (11), 113 (18), 99 (27), 85 (65), 71 (80), 57 (100), 43 (53).

## Results

### Discrimination of Different CHC Profiles

We tested workers of different sub-colonies in one-on-one encounters and used aggressive responses as a measure of the workers’ categorization of the encounter worker as either “nestmate” or “non-nestmate.” Our manipulations allowed us to address the question as to whether aggression is related either to chemical similarity, or to the worker’s experience with the different CHC profiles (familiar and unfamiliar CHC profiles).

The CHC profile of the sub-colony where both the linear and the methylated hydrocarbons were added (sub-colony AB), is more similar to the CHC profile of the sub-colony where only the methylated hydrocarbon was added (sub-colony A) than from that of the sham-treated sub-colony (sub-colony 0).

In almost all one-on-one encounters (353 of 360) between focal workers and stimulus workers, we were able to classify the behavior of the focal worker as aggressive or non-aggressive. In seven encounters, we were unable to classify the focal worker’s behavior because one of the two workers escaped the arena by climbing the wall, thus avoiding an encounter.

**Figure 2:**
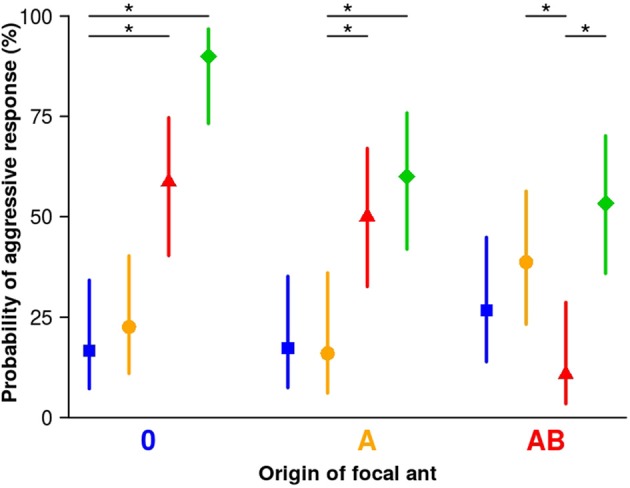
Aggression of focal workers towards stimulus workers from different sub-colonies. Focal workers from all sub-colonies rarely showed aggression towards stimulus workers from their own sub-colony (matching colors), or from the sham-treated sub-colony 0 (blue squares). In contrast, a significant number of focal workers from all sub-colonies showed aggression towards stimulus workers from sub-colony C (green diamonds). Focal workers from sub-colony 0 significantly more often showed aggression towards stimulus workers from sub-colony AB (red triangles) but not so towards stimulus workers from sub-colony A (orange circles). Focal workers of sub-colony A showed aggression significantly more often towards stimulus workers from sub-colony AB (red triangles) than towards stimulus workers from their own sub-colony. Focal workers of sub-colony AB showed aggression significantly more often towards stimulus workers from sub-colony A (orange circle) than towards stimulus workers from their own sub-colony. Symbols are fitted values from the binomial generalized linear model (GLM; model 2) and vertical lines represent the 95% credible intervals based on Bayesian statistics. Between 25 and 31 workers from each group were tested. Asterisks indicate levels of certainty above 99% for a difference between matching (from same sub-colony) and mismatching (from different sub-colonies) pairs.

We rarely found aggression in encounters between focal and stimulus workers from matching (same) sub-colonies (10.7%–16.7%, Figure 2), indicating that coherence within the sub-colonies is maintained.

The majority of focal workers from the manipulated sub-colony A also did not show aggression to stimulus workers from the sham-treated sub-colony (sub-colony 0; Figure 2 blue; 17.2%), which shows that the original colony odor is still accepted. Focal workers from sub-colony AB more often (level of certainty: 93%) responded with aggression towards stimulus workers from sub-colony 0 (26.7%) than towards stimulus workers from their own sub-colony (10.7%), indicating that about 3/4 of the workers of sub-colony 0 are still accepted.

The percentage of focal workers from sub-colony 0 that showed aggression towards stimulus workers treated with A was also low (22.6%), and we found little support that it differs from workers’ responses towards stimulus workers from their own sub-colony (level of certainty only 71.6%). It seems that the presence of component A (linear alkane C30) in stimulus workers does not signify non-nestmate status to focal workers from sub-colony 0. By contrast, focal workers from sub-colony 0 and sub-colony A displayed aggression towards stimulus workers from sub-colony AB (42% and 34% increase of aggression, respectively; levels of certainty >99% in both cases). Thus, adding the two components A and B (C30 and 5-MeC27) to the CHC profile of stimulus workers induces aggressive responses in focal workers from sub-colonies in which none (sub-colony 0) or only one of the two components (C30 in sub-colony A) was used for manipulation of the colony odor. Furthermore, focal workers from sub-colony AB significantly more often responded with aggression towards stimulus workers from sub-colony A (38.7%) than towards stimulus workers from their own sub-colony (10.7%). In this test scenario, the lack of component B in the CHC profile of the stimulus workers induces more often aggressive behavior in focal workers of sub-colony AB compared to encounters with their current nestmates (level of certainty >99%). With a certainty of 83.8%, workers of sub-colony AB are more often aggressive towards workers from sub-colony A than towards workers from sub-colony 0. Thus, both the presence of an additional component and the lack of a specific component can alter the CHC profile of a nestmate to that of a non-nestmate.

In addition, we tested focal workers from all three sub-colonies (0, A, AB) against stimulus workers from another sub-colony that was treated with a methylated hydrocarbon at high concentration (11-MeC27, sub-colony C), to quantify aggression against an excessively manipulated CHC profile that should easily be recognized as unfamiliar and belonging to non-nestmates. All focal workers, irrespective of which sub-colony they were selected from, showed significant aggression towards stimulus workers from sub-colony C (levels of certainty >99%, Figure 2 green).

### Manipulation of CHC Profiles

**Figure 3:**
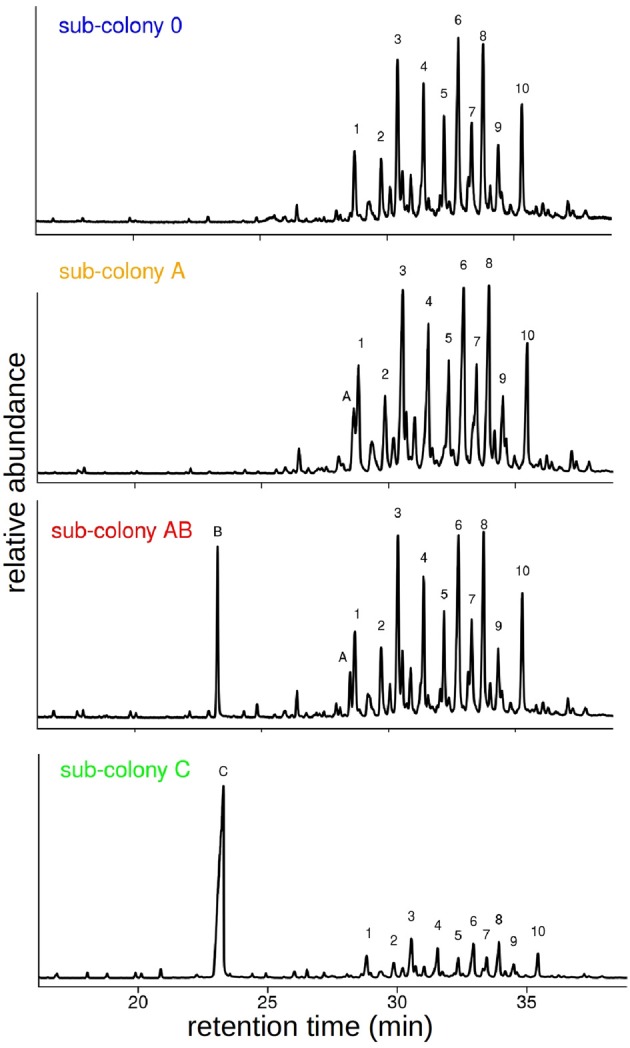
Manipulation of CHC profiles of the different sub-colonies. Example chromatograms of single workers from sub-colonies 0, A, AB and C. Components are indicated by letters and numbers: A: C30, B: Me-5C27, C: Me-11C27, 1: 3,7-di-methyl- and 3,9-di-methylnonacosane, 2: 4-methyltriacontane, 3: 4,10-di-methyl- and 4,8-di-methyltriacontane, 4: 9-methylhentriacontane and unknown, 5: 5,9-di-methylhentriacontane, 6: 8-methyl- and 12-methyldotriacontane, 7: 3,7,11-tri-methylhentriacontane, 8 + 9 + 10: unknown. Note, in these examples the two components A and B are integrated in different amounts into the CHC profile.

Following the behavioral experiments, we collected workers and analyzed their CHC profiles in order to verify that the synthetic hydrocarbons had been transferred to their cuticle by our manipulation. GC-MS analyses of cuticular washes from a total of 38 individual workers revealed that our chemical manipulation of the colony odor was successful. CHC extracts of individuals contained the same prominent CHCs as the sham-treated sub-colony (sub-colony 0) plus one or two additional hydrocarbons depending on the manipulation (Table 1). The additional hydrocarbons A and B were detected as distinct peaks in the chromatograms at retention times where no components of the original CHC profile of *C. floridanus* appear (Figure 3). Based on the GC-MS data, we cannot rule out that we potentially collected synthetic hydrocarbons that were taken up by the ants but not presented as part of the CHCs on the cuticle.

**Table 1:** Relative amounts (mean ± SD) of the 10 most abundant cuticular hydrocarbons (CHCs) in extracts obtained from individual workers (normalized to total peak area).

Peak	Sub-colony 0 *n* = 11	Sub-colony A *n* = 12	Sub-colony AB *n* = 8	Sub-colony C *n* = 7
A	0.2 ± 0.4	8.9 ± 6.9	4.8 ± 5.1	0.8 ± 1.3
B	0 ± 0	0 ± 0	11.7 ± 6.9	0 ± 0
C	0 ± 0	0 ± 0	0 ± 0	174.4 ± 115.5
1	6.5 ± 0.8	8.1 ± 0.5	7.8 ± 0.8	7.4 ± 0.8
2	5.3 ± 1.3	5.8 ± 1.1	5.8 ± 0.9	6.4 ± 1.1
3	14.1 ± 0.7	14.6 ± 0.6	14.7 ± 0.6	14.5 ± 0.8
4	10.8 ± 0.7	10.9 ± 0.8	11.1 ± 0.4	10.9 ± 0.6
5	6.9 ± 0.3	7 ± 0.4	6.8 ± 0.2	6.6 ± 0.2
6	18 ± 2.7	17.7 ± 2.7	17.2 ± 1.1	18.4 ± 1.9
7	8.1 ± 1.2	7.9 ± 1.3	7.8 ± 0.8	7.2 ± 0.5
8	15.4 ± 1.4	14.1 ± 1.7	14.9 ± 1.3	14.5 ± 0.9
9	6 ± 0.7	5.6 ± 0.7	5.4 ± 0.2	5.6 ± 0.4
10	9.1 ± 1.0	8.3 ± 1	8.6 ± 0.8	8.4 ± 0.5

*Peak indices as marked in Figure 3*.

**Figure 4:**
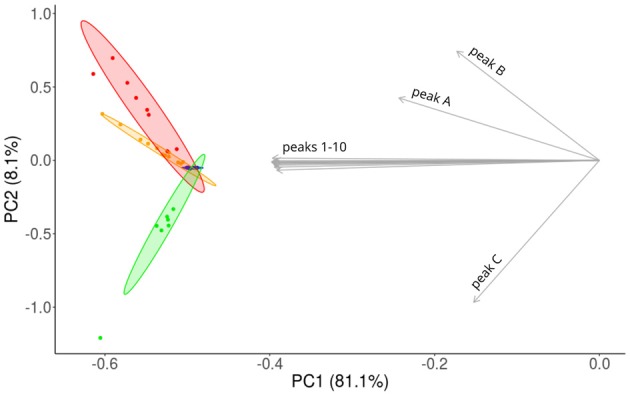
Principal component analysis (PCA) of CHC profiles obtained from single workers. Workers differ systematically, depending on treatment and are aligned on different axes. PCA reveals that the amounts of components integrated into the CHC profile within particular sub-colonies are variable. Ellipses represent 95% confidence areas. Blue: 11 workers of sub-colony 0, yellow: 12 workers of sub-colony A, red: eight workers of sub-colony AB, green: seven workers of sub-colony C. Gray arrows indicate loadings for the same 13 hydrocarbons as in Table 1.

We visualized the similarities of CHC profiles between the sub-colonies by a PCA. CHC profiles of workers were spread along PC1, whereas workers from sub-colonies A and AB were separated from workers of sub-colony C along PC2 (Figure 4). Chemically, the most homogeneous group were the workers from sub-colony 0, and on average the workers from sub-colony A were chemically more similar than workers from sub-colony AB, compared to workers from sub-colony 0.

## Discussion

We investigated whether workers use commonalities found in CHC profiles to recognize nestmates, or whether nestmate recognition is based on the generalization of distinct CHC profiles that can be discriminated by workers. We successfully manipulated the colony odor of different sub-colonies by adding one or two different hydrocarbons that were not present in the original CHC profiles in *C. floridanus*, at levels similar (for component A and B, not for component C) to the amounts of the original hydrocarbons. This allowed us to test the effects of adding novel components to the profile, and also, to test the effect of the lack of one component of an otherwise very similar CHC profile. We found that workers can adjust their nestmate recognition to novel, manipulated CHC profiles, but still accept workers as nestmates that carry the previous CHC profile. Furthermore, we show that additional components as well as the lack of a component in the CHC profile can be discriminated and can render a label as belonging to non-nestmates. Workers having both the linear and the methyl-branched hydrocarbon in their novel CHC profile perceive workers having only the linear hydrocarbon, but not the methylated hydrocarbon, as being non-nestmates. Thus, our results support the hypothesis that workers can recognize nestmates with different CHC profiles in a multiple-template recognition process and categorize them as all belonging to nestmates. Whether they discriminate between the different nestmate CHC profiles remains to be tested.

### Discrimination of CHC Profiles

For our manipulation of the CHC profiles, we used two methyl-branched and one straight-chain hydrocarbon, knowing that hydrocarbons containing methyl groups may be more important as recognition cues compared to unbranched alkanes (Dani et al., 2001; Guerrieri et al., 2009; Martin S. and Drijfhout F., 2009), but see (Akino et al., 2004; Greene and Gordon, 2007). Indeed, we found that adding a linear alkane (C30) to the CHC profile did not significantly affect nestmate recognition in workers that did not have this component in their own colony odor. We hypothesize that components of the CHC profile can be insignificant or important recognition cues, based on whether their presence has predictive power for discrimination extending the concept of key recognition cues in CHC profiles (Dani et al., 2001; Guerrieri et al., 2009; Martin S. J. and Drijfhout F. P., 2009; van Zweden et al., 2014).

Our observations that after manipulation of the colony odor, the novel as well as the previous CHC profile is accepted for nestmate recognition led to the assumption that the underlying mechanism is a widening of the acceptance range of the template for nestmate labels (Meskali et al., 1995; Leonhardt et al., 2007; Guerrieri et al., 2009). In one setting, aggression against workers with the previous CHC profile was comparatively high (workers from sub-colony AB towards workers of sub-colony 0). We do not know the reason for this, but we consider the variation in mean aggression probability across the different groups tested against their previous CHC profile as a result of small sample size.

The results presented here cannot be explained in the framework of diverse labels that are recognized with a unifying-template. A unifying-template would be based on commonalities found in the majority of all nestmate CHC profiles and their corresponding neural representation, which led to the concept of “inclusion theory” (Guerrieri et al., 2009; Bos et al., 2012, 2013). However, in one of our test scenarios (workers from sub-colony AB encountering workers from sub-colony A), the inclusion criterion with respect to the chemical composition of the CHC profiles was fulfilled. We showed that workers were still able to discriminate based on CHC profiles that contain all except one of the components, compared to their own (manipulated) colony odor. Thus, with respect to the labels, i.e., composition of CHC profiles, the workers’ discrimination cannot be explained by the inclusion theory, because additional components were not more important than missing components for nestmate recognition, as had previously been suggested (Guerrieri et al., 2009).

### Neural Mechanisms for Label-Template Matching and Novel Template Formation

**Figure 5:**
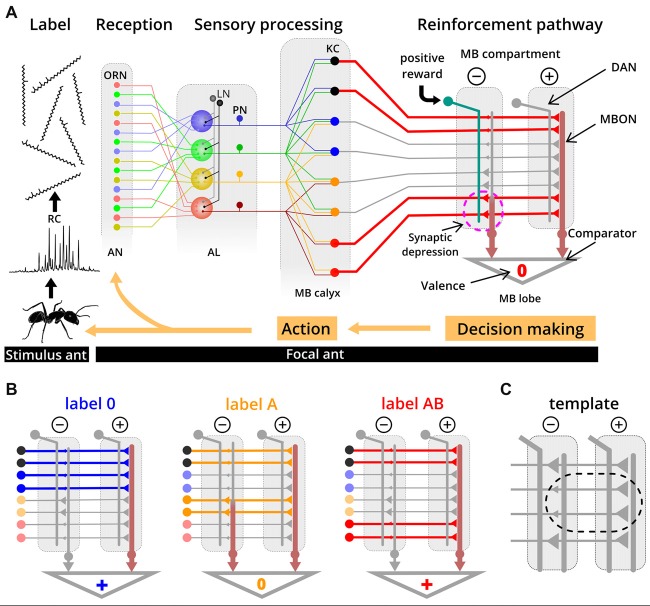
Simplified circuit model proposed for template formation and label-template matching. **(A)** Recognition cues (RCs) from a stimulus ant are received by broadly tuned olfactory receptor neurons (ORNs) on the antennae (AN) of the evaluating ant. The odor-induced activity is reformatted along the olfactory pathway through the antennal lobe (AL) and the projection neurons (PN), resulting in label-specific activation patterns of Kenyon cells (KCs) in the mushroom body (MB). In this example, label AB induced activity in KCs is indicated as thick red lines. During learning of a novel label (e.g., label AB), a reward results in activation of dopaminergic neurons (DAN) in one of the two compartments. Coincident activity of DAN and KC in this compartment results in synaptic depression between the KC and the MBON. Based on the relative output strength from different MB compartments, a positive, neutral or negative valence is provided, a decision is made and the focal ant eventually responds with a behavioral action towards the stimulus ant. In this example, label AB activates a specific set of KC, during a learning process. The synapses from KCs that are also active when the label 0 is presented are already depressed (from previous learning events), whereas synapses of label AB-specific KCs undergo depression by coincident activity in DAN (dashed circle). **(B)** Examples of synaptic transmission between KCs and MBONs at the two different compartments after learning label 0 and label AB as nestmate. All three labels (label 0, A and AB) activate different sets and the same number of KCs (colored thick lines indicate activity; gray thin lines indicated no activity). Due to weaker synapses between KC and MBON in the negative compartment, label 0 and label AB have an overall positive valence, while label A has a neutral valence. Functional connectivity of DAN and MBON in the MB is derived from studies on *Drosophila* (Aso et al., 2014). KC color code: black, unspecific KC for colony odors; blue, label 0 specific KC; orange, label A specific KC, red, label AB specific KC. LN: local interneurons. **(C)** Based on our proposed model, the template is implemented as reduced synaptic strength between nestmate label-specific KCs and MBON in the negative MB compartment compared to the positive compartment.

Ants learn about their colony odor during early adult life (Carlin and Hölldobler, 1983; Errard, 1994) and of novel labels later in life (Guerrieri et al., 2009), as is the case in our experiments. Because they match labels with a neural template, we propose that this takes place in the mushroom body (MB)—a brain area which is important for learning and memory (Heisenberg, 2003; Giurfa, 2007). The components of a label are detected by broadly tuned olfactory receptor neurons (ORNs; Sharma et al., 2015) which project to the antennal lobe (AL) where ORNs that express the same olfactory receptors coalesce in the same functional units (Couto et al., 2005; Zube et al., 2008; Figure 5A). Here, the label-specific activity patterns are reformatted and conveyed via projection neurons (PNs) to higher brain centers, such as the MB (Deisig et al., 2010; Brandstaetter and Kleineidam, 2011). At the calyces of the MB, PNs synapse onto Kenyon cells (KCs) which transform dense and overlapping activity patterns into sparse and much less overlapping activity patterns (Perez-Orive et al., 2002; Szyszka et al., 2005). In seeking for the neural substrate of templates, we adopt the current knowledge on evaluation and learning processes in the MB of the fruit fly *Drosophila* (Aso et al., 2014; Cohn et al., 2015; Hige et al., 2015a,b) and of the honey bee (Strube-Bloss et al., 2011, 2016) in order to propose the following mechanism for label-template matching.

Many KCs (~2,000 in *Drosophila* and ~170,000 in honey bees; Witthöft, 1967; Aso et al., 2014) synapse onto a few output neurons (MBON; 34 in *Drosophila* and ~400 in honey bees; Rybak and Menzel, 1993; Tanaka et al., 2008) in different compartments of the MB. One compartment of the MB is positive and another, corresponding one is negative, each of both contains an extrinsic MBON. We postulate that a label will be associated with positive or negative valence when the strength of MBON activity from different compartments becomes unbalanced (Figure 5B). Unbalanced activity across MBONs results from compartment-specific differences in synaptic transmission from KCs to MBONs, and we assume that synaptic transmission is reduced in the compartment providing negative valence (Hige et al., 2015a). A lower synaptic transmission is restricted to those synapses of KCs that are activated by nestmate label. Based on this framework, the neural template would be implemented as weaker synaptic strength between KC and MBON in the negative compartment compared to the positive compartment (Figure 5C). Plasticity in KC synapses can be induced by coincident activity of compartment-specific dopaminergic neurons (DANs), which predominantly leads to synaptic depression (honey bee: Mauelshagen, 1993; Grünewald, 1999; *Drosophila*: Hige et al., 2015a) but can additionally lead to recruitment of MBON activity (see Okada et al., 2007; Strube-Bloss et al., 2011, 2016). Template formation through associative learning requires the activation of the DAN pathway by a reward (e.g., social interaction or food) and possibly also by recurrent activation of DANs from MBONs (Zhao et al., 2018). Experiencing a novel label that eventually becomes valid for nestmates would lead to synaptic depression at all synapses of KCs that are activated by this novel label in the compartment providing negative valence. Synaptic depression due to distinct nestmate labels could occur on the same MBON and we assume that a high specificity to different labels is realized by the large number of KCs and their sparse activity patterns for different labels. Specifically, we assume that there are KCs which show the response property of responding to one stimulus (e.g., label A), but not to a similar stimulus where only one additional component is added (e.g., label AB; Figure 5B). Note that in *Drosophila* such KCs (responding to one component but not in combination with a second component) have not been described yet (Campbell et al., 2013). However, studies in *Drosophila* and honey bees suggest that such synthetic processing of information about components occurs in the AL (Joerges et al., 1997; Silbering and Galizia, 2007; Silbering et al., 2008; Deisig et al., 2010). Moreover, associative odor learning alters the activity patterns in functional units of the AL which enhances the difference between mixtures and their components (Faber et al., 1999; Daly et al., 2004; Fernandez et al., 2009; Rath et al., 2011). For our study, this implies modification of AL activity patterns for the label with one additional component (label A), thereby separating it from the activity pattern for the label with the same and another component added (label AB). Our result that the label with one additional component is “strange” only for those workers that learned the label with two additional components as being valid, could also be explained by synthetic processing.

This framework for a template-formation mechanism could also explain the ability of workers to discriminate between several task-associated label from the same colony (Bonavita-Cougourdan et al., 1993; Greene and Gordon, 2003), if one assumes that different task-related labels would be encoded by different MBONs.

### Significance for Colony Organization

Our proposed mechanism of multiple-template for nestmate recognition changes the paradigm on what information about colony members is acquired and how it is used. Under a previous hypothesis (the one-template system), membership initially is recognized and subsequently additional information such as cues about recent task performance is processed. Instead, our data suggests that recognition cues of colony members can be perceived and classified as belonging to distinct entities, and different labels can be attributed independently with more than one attribute of e.g., “nestmate and forager” or “nestmate and nurse.” Such a partitioned nestmate recognition system is highly selective for different labels while remaining flexible enough to accommodate developing differences due to e.g., task engagement, seasonality or, diet.

Because the proposed associations in the multiple-template system takes place along parallel pathways in the MB, it requires less time for processing compared to a sequential categorization with additional information such as task engagement in a one-template system. Workers can recognize nestmates very quickly (Stroeymeyt et al., 2010), and conversely, fast reactions against non-nestmates are probably selected for because a delayed response increases the risk of being injured, and the first to react will probably have an advantage in any ensuing conflict. Because individual experience shapes the templates, we expect that template formation is an individual process and colony members probably have different templates, even for the same label. Inter-individual differences in nestmate recognition have been discussed as one potential mechanism for a reliable defensive response of the collective (Esponda and Gordon, 2015).

A further consequence of the individual reinforcement during template formation and maintenance is that frequent interactions within a task-group can adjust templates accordingly. For example, workers involved in brood care interact more with nurses, and thereby may tune their corresponding template for “nestmate and nurse” more than workers which are engaged in a different task somewhere else. This may result in experience-dependent improvement for detection of additional cues within the label that characterize the task-group. In this scenario, nurse workers know more about nurses, foraging workers more about foragers, guards probably more about foragers, and so on. Indeed, workers show spatial fidelity within the nest (Mersch et al., 2013; Tschinkel and Hanley, 2017) and although task allocation is flexible, workers tend to reengage in the same task over longer periods of time (Beshers and Fewell, 2001; Jeanson and Weidenmüller, 2014). A partitioned nestmate recognition system with multiple highly specific templates can contribute to task allocation when distinct sensory experience affects future decisions on task engagements (Sendova-Franks and Franks, 1995; Pamminger et al., 2014).

### The Concept of Label-Template Matching

Describing the process of nestmate recognition in terms of label-template matching originally was intended to provide a conceptual framework for studies on kin recognition. The assumption that only one neural template is necessary and implemented probably was stimulated by the findings of systematic differences in CHC profiles among and low variability within colonies. Later, it turned out that CHC profiles are dynamic within a colony over time, as well as there being differences among colony members according to their functional roles, which challenges the one-template hypothesis. However, the question as to whether workers may employ multiple templates has never been addressed systematically before. In addition, the complexity of the CHC profiles and the rapid discrimination ability of workers resulted in research focusing on possible data reduction mechanisms from detection to perception, such as the inclusion theory, rather than acknowledging the high efficiency and discriminatory abilities of the highly evolved olfactory system of workers in assessing and categorizing complex CHC blends.

We suggest that using the term label-template matching is still justified for highlighting the chemical uniformation within colonies, given that all colony members can recognize their own colony odor. However, when addressing the underlying mechanisms of nestmate recognition, this simplification is more misleading than informative because it does not adequately describe a partitioned nestmate recognition system that allows fine-tuned categorization of chemically distinct nestmates.

## Glossary

CHC profile—Cuticular hydrocarbons that are present on the insect cuticle. Commonly used solvents to extract these hydrocarbons include nonpolar solvents such as pentane, hexane, dichloromethane and isooctane.

- Recognition cues—Components of the CHC profile that can be detected and are used for nestmate recognition.
- Label—The sum of recognition cues in a CHC profile.
- Colony odor—The sum of all chemical components that are present on the cuticle of colony members. It includes nestmate recognition cues and other components not used for nestmate recognition. Note: this commonly used definition refers to the chemical environment and not to the perception and evaluation of chemical components.
- Template—A neural representation of a nestmate label.
- Reception—Detection of chemical components (odorants) by ORNs which encode odorants as changes in timing/rate of action potentials.
- Activity pattern—Parallel pathways in the olfactory system that relay information about the stimulus to functional units, such as glomeruli in the AL or KCs in the MB. The activity across these functional units (assembly code) is stimulus specific and can be recorded as activity patterns over time.
- Representation—An activity pattern that is attributed with a value (learned or innate) and thereby becomes meaningful. The representation of chemical stimuli (odorants) in the brain allows the perception of odors.
- Generalization—Chemical stimuli that can be discriminated are evaluated or categorized as belonging to a common entity.

## Author Contributions

CK, MH and SN conceived and designed the experiments. MH performed the experiments, collected the data on workers’ behavior and the extracts for GC-MS analysis. SN conducted the formal analysis of the behavioral data. SN and MH analyzed the GC-MS data. JM synthesized the components 5-MeC27 and 11-MeC27 for the study. SN prepared the first draft of the manuscript. CK revised it critically for important intellectual content. Final version of the manuscript was approved by all authors.

## Conflict of Interest Statement

The authors declare that the research was conducted in the absence of any commercial or financial relationships that could be construed as a potential conflict of interest.

## References

[B1] Akino T. Yamamura K. Wakamura S. Yamaoka R. (2004). Direct behavioral evidence for hydrocarbons as nestmate recognition cues in *Formica japonica* (Hymenoptera: Formicidae). Appl. Entomol. Zool. 39, 381–387. 10.1303/aez.2004.381

[B2] Aso Y. Hattori D. Yu Y. Johnston R. M. Iyer N. A. Ngo T. T.. (2014). The neuronal architecture of the mushroom body provides a logic for associative learning. ELife 3:e04577. 10.7554/eLife.04577 25535793 PMC4273437

[B3] Beshers S. N. Fewell J. H. (2001). Models of division of labor in social insects. Annu. Rev. Entomol. 46, 413–440. 10.1146/annurev.ento.46.1.413 11112175

[B4] Bitterman M. E. Menzel R. Fietz A. Schäfer S. (1983). Classical-conditioning of proboscis extension in honeybees (*Apis mellifera*). J. Comp. Psychol. 97, 107–119. 10.1037/0735-7036.97.2.107 6872507

[B5] Bonavita-Cougourdan A. Clement J. L. Lange C. (1993). Functional subcaste discrimination (foragers and brood-tenders) in the ant *Camponotus vagus* Scop.: polymorphism of cuticular hydrocarbon patterns. J. Chem. Ecol. 19, 1461–1477. 10.1007/bf00984890 24249176

[B6] Bos N. (2014). Asymmetry in olfactory generalization and the inclusion criterion in ants. Commun. Integr. Biol. 7:e29163. 10.4161/cib.29163 25346797 PMC4203582

[B7] Bos N. d’Ettorre P. (2012). Recognition of social identity in ants. Front. Psychol. 3:83. 10.3389/fpsyg.2012.00083 22461777 PMC3309994

[B8] Bos N. d’Ettorre P. Guerrieri F. J. (2013). Chemical structure of odorants and perceptual similarity in ants. J. Exp. Biol. 216, 3314–3320. 10.1242/jeb.087007 23685976

[B9] Bos N. Dreier S. Jorgensen C. G. Nielsen J. Guerrieri F. J. d’Ettorre P. (2012). Learning and perceptual similarity among cuticular hydrocarbons in ants. J. Insect Physiol. 58, 138–146. 10.1016/j.jinsphys.2011.10.010 22067290

[B11] Brandstaetter A. S. Endler A. Kleineidam C. J. (2008). Nestmate recognition in ants is possible without tactile interaction. Naturwissenschaften 95, 601–608. 10.1007/s00114-008-0360-5 18350268

[B10] Brandstaetter A. S. Kleineidam C. J. (2011). Distributed representation of social odors indicates parallel processing in the antennal lobe of ants. J. Neurophysiol. 106, 2437–2449. 10.1152/jn.01106.2010 21849606

[B12] Brandstaetter A. S. Röessler W. Kleineidam C. J. (2011). Friends and foes from an ant brain’s point of view—neuronal correlates of colony odors in a social insect. PLoS One 6:e21383. 10.1371/journal.pone.0021383 21731724 PMC3121771

[B100] Buckley S. B. (1866). Descriptions of new species of North American Formicidae. Proc. Entomol. Soc. Phila. 6, 152–172.

[B13] Campbell R. A. A. Honegger K. S. Qin H. Li W. Demir E. Turner G. C. (2013). Imaging a population code for odor identity in the *Drosophila* mushroom body. J. Neurosci. 33, 10568–10581. 10.1523/JNEUROSCI.0682-12.2013 23785169 PMC3685844

[B14] Carlin N. F. Hölldobler B. (1983). Nestmate and kin recognition in interspecific mixed colonies of ants. Science 222, 1027–1029. 10.1126/science.222.4627.1027 17776248

[B15] Cohn R. Morantte I. Ruta V. (2015). Coordinated and compartmentalized neuromodulation shapes sensory processing in *Drosophila*. Cell 163, 1742–1755. 10.1016/j.cell.2015.11.019 26687359 PMC4732734

[B16] Couto A. Alenius M. Dickson B. J. (2005). Molecular, anatomical, and functional organization of the *Drosophila* olfactory system. Curr. Biol. 15, 1535–1547. 10.1016/j.cub.2005.07.034 16139208

[B17] Crozier R. H. Dix M. W. (1979). Analysis of 2 genetic models for the innate components of colony odor in social Hymenoptera. Behav. Ecol. Sociobiol. 4, 217–224. 10.1007/bf00297645

[B18] Daly K. C. Christensen T. A. Lei H. Smith B. H. Hildebrand J. G. (2004). Learning modulates the ensemble representations for odors in primary olfactory networks. Proc. Natl. Acad. Sci. U S A 101, 10476–10481. 10.1073/pnas.0401902101 15232007 PMC478594

[B19] Dani F. R. Jones G. R. Destri S. Spencer S. H. Turillazzi S. (2001). Deciphering the recognition signature within the cuticular chemical profile of paper wasps. Anim. Behav. 62, 165–171. 10.1006/anbe.2001.1714

[B20] Deisig N. Giurfa M. Sandoz J. C. (2010). Antennal lobe processing increases separability of odor mixture representations in the honeybee. J. Neurophysiol. 103, 2185–2194. 10.1152/jn.00342.2009 20181736

[B21] Errard C. (1994). Long-term-memory involved in nestmate recognition in ants. Anim. Behav. 48, 263–271. 10.1006/anbe.1994.1240

[B22] Esponda F. Gordon D. M. (2015). Distributed nestmate recognition in ants. Proc. Biol. Sci. 282:20142838. 10.1098/rspb.2014.2838 25833853 PMC4426612

[B23] Faber T. Joerges J. Menzel R. (1999). Associative learning modifies neural representations of odors in the insect brain. Nat. Neurosci. 2, 74–78. 10.1038/4576 10195183

[B24] Fernandez P. C. Locatelli F. F. Person-Rennell N. Deleo G. Smith B. H. (2009). Associative conditioning tunes transient dynamics of early olfactory processing. J. Neurosci. 29, 10191–10202. 10.1523/JNEUROSCI.1874-09.2009 19692594 PMC2756734

[B25] Fielde A. (1903). Artificial mixed nests of ants. Biol. Bull. 5, 320–325. 10.2307/1535842

[B26] Gadau J. Heinze J. Hölldobler B. Schmid M. (1996). Population and colony structure of the carpenter ant *Camponotus floridanus*. Mol. Ecol. 5, 785–792. 10.1111/j.1365-294x.1996.tb00374.x 8981768

[B27] Gelman A. Hill J. (2007). Data Analysis Using Regression and Multilevel/Hierarchial Models. New York, NY: Cambridge University Press.

[B28] Gerber B. Geberzahn N. Hellstern F. Klein J. Kowalksy O. Wüstenberg D. (1996). Honey bees transfer olfactory memories established during flower visits to a proboscis extension paradigm in the laboratory. Anim. Behav. 52, 1079–1085. 10.1006/anbe.1996.0255

[B29] Giurfa M. (2007). Behavioral and neural analysis of associative learning in the honeybee: a taste from the magic well. J. Comp. Physiol. A Neuroethol. Sens. Neural Behav. Physiol. 193, 801–824. 10.1007/s00359-007-0235-9 17639413

[B30] Greene M. J. Gordon D. M. (2003). Social insects: cuticular hydrocarbons inform task decisions. Nature 423:32. 10.1038/423032a 12721617

[B31] Greene M. J. Gordon D. M. (2007). Structural complexity of chemical recognition cues affects the perception of group membership in the ants *Linephithema humile* and *Aphaenogaster cockerelli*. J. Exp. Biol. 210, 897–905. 10.1242/jeb.02706 17297148

[B32] Grünewald B. (1999). Physiological properties and response modulations of mushroom body feedback neurons during olfactory learning in the honeybee, *Apis mellifera*. J. Comp. Physiol. 185, 565–576. 10.1007/s003590050417

[B33] Guerrieri F. J. Nehring V. Jorgensen C. G. Nielsen J. Galizia C. G. d’Ettorre P. (2009). Ants recognize foes and not friends. Proc. Biol. Sci. 276, 2461–2468. 10.1098/rspb.2008.1860 19364750 PMC2690455

[B34] Heisenberg M. (2003). Mushroom body memoir: from maps to models. Nat. Rev. Neurosci. 4, 266–275. 10.1038/nrn1074 12671643

[B35] Hige T. Aso Y. Modi M. N. Rubin G. M. Turner G. C. (2015a). Heterosynaptic plasticity underlies aversive olfactory learning in *Drosophila*. Neuron 88, 985–998. 10.1016/j.neuron.2015.11.003 26637800 PMC4674068

[B36] Hige T. Aso Y. Rubin G. M. Turner G. C. (2015b). Plasticity-driven individualization of olfactory coding in mushroom body output neurons. Nature 526, 258–262. 10.1038/nature15396 26416731 PMC4860018

[B37] Hölldobler B. Wilson E. O. (1990). The Ants. Cambridge, MA: Belknap Press of Harvard University Press.

[B38] Holmes W. Sherman P. (1983). Kin recognition in animals. Amer. Sci. 71, 46–55.

[B39] Jeanson R. Weidenmüller A. (2014). Interindividual variability in social insects—proximate causes and ultimate consequences. Biol. Rev. Camb. Philos. Soc. 89, 671–687. 10.1111/brv.12074 24341677

[B40] Joerges J. Kuttner A. Galizia C. G. Menzel R. (1997). Representations of odours and odour mixtures visualized in the honeybee brain. Nature 387, 285–288. 10.1038/387285a0

[B41] Jutsum A. R. Saunders T. S. Cherrett J. M. (1979). Intraspecific aggression in the leaf-cutting ant *Acromyrmex octospinosus*. Anim. Behav. 27, 839–844. 10.1016/0003-3472(79)90021-6

[B42] Kaib M. Eisermann B. Schoeters E. Billen J. Franke S. Francke W. (2000). Task-related variation of postpharyngeal and cuticular hydrocarbon compositions in the ant *Myrmicaria eumenoides*. J. Comp. Physiol. A 186, 939–948. 10.1007/s003590000146 11138794

[B43] Kleineidam C. J. Heeb E. L. Neupert S. (2017). Social interactions promote adaptive resource defense in ants. PLoS One 12:e0183872. 10.1371/journal.pone.0183872 28910322 PMC5598949

[B44] Lacy R. C. Sherman P. W. (1983). Kin recognition by phenotype matching. Am. Nat. 121, 489–512. 10.1086/284078

[B45] Lahav S. Soroker V. Hefetz A. Vander Meer R. K. (1999). Direct behavioral evidence for hydrocarbons as ant recognition discriminators. Naturwissenschaften 86, 246–249. 10.1007/s001140050609

[B46] Lenoir A. Hefetz A. Simon T. Soroker V. (2001). Comparative dynamics of gestalt odour formation in two ant species *Camponotus fellah* and *Aphaenogaster senilis* (Hymenoptera: Formicidae). Physiol. Entomol. 26, 275–283. 10.1046/j.0307-6962.2001.00244.x

[B47] Leonhardt S. D. Brandstaetter A. S. Kleineidam C. J. (2007). Reformation process of the neuronal template for nestmate-recognition cues in the carpenter ant *Camponotus floridanus*. J. Comp. Physiol. A Neuroethol. Sens. Neural Behav. Physiol. 193, 993–1000. 10.1007/s00359-007-0252-8 17639411

[B48] Lockey K. H. (1988). Lipids of the insect cuticle—origin, composition and function. Comp. Biochem. Phys. B 89, 595–645. 10.1016/0305-0491(88)90305-7

[B49] Martin S. Drijfhout F. (2009). A review of ant cuticular hydrocarbons. J. Chem. Ecol. 35, 1151–1161. 10.1007/s10886-009-9695-4 19866237

[B50] Martin S. J. Drijfhout F. P. (2009). Nestmate and task cues are influenced and encoded differently within ant cuticular hydrocarbon profiles. J. Chem. Ecol. 35, 368–374. 10.1007/s10886-009-9612-x 19263166

[B51] Mauelshagen J. (1993). Neural correlates of olfactory learning paradigms in an identified neuron in the honeybee brain. J. Neurophysiol. 69, 609–625. 10.1152/jn.1993.69.2.609 8459289

[B52] Mersch D. P. Crespi A. Keller L. (2013). Tracking individuals shows spatial fidelity is a key regulator of ant social organization. Science 340, 1090–1093. 10.1126/science.1234316 23599264

[B53] Meskali M. Provost E. Bonavita-Cougourdan A. Clement J. L. (1995). Behavioral-effects of an experimental change in the chemical signature of the ant *Camponotus vagus* (scop). Insect. Soc. 42, 347–358. 10.1007/bf01242163

[B54] Morel L. Blum M. (1988). Nestmate recognition in *Camponotus floridanus* callow worker ants—are sisters or nestmates recognized? Anim. Behav. 36, 718–725. 10.1016/s0003-3472(88)80154-4

[B55] Okada R. Rybak J. Manz G. Menzel R. (2007). Learning-related plasticity in PE1 and other mushroom body-extrinsic neurons in the honeybee brain. J. Neurosci. 27, 11736–11747. 10.1523/JNEUROSCI.2216-07.2007 17959815 PMC6673233

[B56] Ozaki M. Wada-Katsumata A. Fujikawa K. Iwasaki M. Yokohari F. Satoji Y.. (2005). Ant nestmate and non-nestmate discrimination by a chemosensory sensillum. Science 309, 311–314. 10.1126/science.1105244 15947139

[B57] Pamminger T. Foitzik S. Kaufmann K. C. Schützler N. Menzel F. (2014). Worker personality and its association with spatially structured division of labor. PLoS One 9:e79616. 10.1371/journal.pone.0079616 24497911 PMC3907378

[B58] Perez-Orive J. Mazor O. Turner G. C. Cassenaer S. Wilson R. I. Laurent G. (2002). Oscillations and sparsening of odor representations in the mushroom body. Science 297, 359–365. 10.1126/science.1070502 12130775

[B59] R Core Team (2017). R: A Language and Environment for Statistical Computing. R Foundation for Statistical Computing. Vienna, Austria Available online at: https://www.R-project.org/

[B60] Rath L. Giovanni Galizia C. Szyszka P. (2011). Multiple memory traces after associative learning in the honey bee antennal lobe. Eur. J. Neurosci. 34, 352–360. 10.1111/j.1460-9568.2011.07753.x 21692886

[B61] Reeve H. (1989). The evolution of conspecific acceptance thresholds. Am. Nat. 133, 407–435. 10.1086/284926

[B62] Rybak J. Menzel R. (1993). Anatomy of the mushroom bodies in the honeybee brain: the neuronal connections of the α-lobe. J. Comp. Neurol. 334, 444–465. 10.1002/cne.903340309 8376627

[B63] Sendova-Franks A. Franks N. (1995). Spatial relationships within nests of the ant *Leptothorax unifasciatus* (Latr.) and their implications for the division of labour. Anim. Behav. 50, 121–136. 10.1006/anbe.1995.0226

[B64] Sharma K. R. Enzmann B. L. Schmidt Y. Moore D. Jones G. R. Parker J.. (2015). Cuticular hydrocarbon pheromones for social behavior and their coding in the ant antenna. Cell Rep. 12, 1261–1271. 10.1016/j.celrep.2015.07.031 26279569

[B65] Silbering A. F. Galizia C. G. (2007). Processing of odor mixtures in the *Drosophila* antennal lobe reveals both global inhibition and glomerulus-specific interactions. J. Neurosci. 27, 11966–11977. 10.1523/JNEUROSCI.3099-07.2007 17978037 PMC6673347

[B66] Silbering A. F. Okada R. Ito K. Galizia C. G. (2008). Olfactory information processing in the *Drosophila* antennal lobe: anything goes? J. Neurosci. 28, 13075–13087. 10.1523/JNEUROSCI.2973-08.2008 19052198 PMC6671615

[B67] Stroeymeyt N. Guerrieri F. J. van Zweden J. S. d’Ettorre P. (2010). Rapid decision-making with side-specific perceptual discrimination in ants. PLoS One 5:e12377. 10.1371/journal.pone.0012377 20808782 PMC2927537

[B68] Strube-Bloss M. F. Nawrot M. P. Menzel R. (2011). Mushroom body output neurons encode odor-reward associations. J. Neurosci. 31, 3129–3140. 10.1523/JNEUROSCI.2583-10.2011 21414933 PMC6623757

[B69] Strube-Bloss M. F. Nawrot M. P. Menzel R. (2016). Neural correlates of side specific odour memory in mushroom body output neurons. Proc. Biol. Sci. 283:20161270. 10.1098/rspb.2016.1270 27974514 PMC5204139

[B70] Szyszka P. Ditzen M. Galkin A. Galizia C. G. Menzel R. (2005). Sparsening and temporal sharpening of olfactory representations in the honeybee mushroom bodies. J. Neurophysiol. 94, 3303–3313. 10.1152/jn.00397.2005 16014792

[B71] Tanaka N. K. Tanimoto H. Ito K. (2008). Neuronal assemblies of the *Drosophila* mushroom body. J. Comp. Neurol. 508, 711–755. 10.1002/cne.21692 18395827

[B72] Tschinkel W. R. Hanley N. (2017). Vertical organization of the division of labor within nests of the Florida harvester ant, *Pogonomyrmex badius*. PLoS One 12:e0188630. 10.1371/journal.pone.0188630 29182686 PMC5705139

[B73] van Zweden J. Pontieri L. Pedersen J. S. (2014). A statistical approach to identify candidate cues for nestmate recognition. Front. Ecol. Evol. 2:73 10.3389/fevo.2014.00073

[B74] Vander Meer R. K. Saliwanchik D. Lavine B. (1989). Temporal changes in colony cuticular hydrocarbon patterns of *Solenopsis invicta* implications for nestmate recognition. J. Chem. Ecol. 15, 2115–2125. 10.1007/BF01207442 24272300

[B75] Wagner D. Brown M. J. F. Broun P. Cuevas W. Moses L. E. Chao D. L.. (1998). Task-related differences in the cuticular hydrocarbon composition of harvester ants, *Pogonomyrmex barbatus*. J. Chem. Ecol. 24, 2021–2037. 10.1023/A:102078150888911545372

[B76] Witthöft W. (1967). Absolute anzahl und verteilung der zellen im hirn der honigbiene. Z. Morphol Tiere 61, 160–184. 10.1007/bf00298776

[B77] Zhao X. Lenek D. Dag U. Dickson B. J. Keleman K. (2018). Persistent activity in a recurrent circuit underlies courtship memory in *Drosophila*. eLife 7:e31425. 10.7554/eLife.31425 29322941 PMC5800849

[B78] Zube C. Kleineidam C. J. Kirschner S. Neef J. Röessler W. (2008). Organization of the olfactory pathway and odor processing in the antennal lobe of the ant *Camponotus floridanus*. J. Comp. Neurol. 506, 425–441. 10.1002/cne.21548 18041786

